# Monte Carlo simulation and film dosimetry for electron therapy in vicinity of a titanium mesh

**DOI:** 10.1120/jacmp.v15i4.4649

**Published:** 2014-07-08

**Authors:** Keyvan Jabbari, Masoumeh Rostampour, Mahnaz Roayaei

**Affiliations:** ^1^ Department of Medical Physics and Engineering School of Medicine, Isfahan University of Medical Sciences Isfahan Iran; ^2^ Department of Radiation Oncology School of Medicine, Isfahan University of Medical Sciences Isfahan Iran

**Keywords:** Monte Carlo, titanium mesh, dosimetry, radiochromic film, electron beam

## Abstract

Titanium (Ti) mesh plates are used as a bone replacement in brain tumor surgeries. In the case of radiotherapy, these plates might interfere with the beam path. The purpose of this study is to evaluate the effect of titanium mesh on the dose distribution of electron fields. Simulations were performed using Monte Carlo BEAMnrc and DOSXYZnrc codes for 6 and 10 MeV electron beams. In Monte Carlo simulation, the shape of the titanium mesh was simulated. The simulated titanium mesh was considered as the one which is used in head and neck surgery with a thickness of 0.055 cm. First, by simulation, the percentage depth dose was obtained while the titanium mesh was present, and these values were then compared with the depth dose of homogeneous phantom with no titanium mesh. In the experimental measurements, the values of depth dose with titanium mesh and without titanium mesh in various depths were measured. The experiments were performed using a RW3 phantom with GAFCHROMIC EBT2 film. The results of experimental measurements were compared with values of depth dose obtained by simulation. In Monte Carlo simulation, as well as experimental measurements, for the voxels immediately beyond the titanium mesh, the change of the dose were evaluated. For this purpose the ratio of the dose for the case with titanium to the case without titanium was calculated as a function of titanium depth. For the voxels before the titanium mesh there was always an increase of the dose up to 13% with respect to the same voxel with no titanium mesh. This is because of the increased back scattering effect of the titanium mesh. The results also showed that for the voxel right beyond the titanium mesh, there is an increased or decreased dose to soft tissues, depending on the depth of the titanium mesh. For the regions before the depth of maximum dose, there is an increase of the dose up to 10% compared to the dose of the same depth in homogeneous phantom. Beyond the depth of maximum dose, there was a 16% decrease in dose. For both 6 and 10 MeV, before the titanium mesh, there was always an increase in dose. If titanium mesh is placed in buildup region, it causes an increase of the dose and could lead to overdose of the adjacent tissue, whereas if titanium mesh is placed beyond the buildup region, it would lead to a decrease in dose compared to the homogenous tissue.

PACS number: 87.53.Bn

## I. INTRODUCTION

Advanced head and neck cancers are often treated through a combination of surgery and postoperative radiotherapy. In some cases in surgery of brain tumors after removing tumor, the skull is refixed using a thin metal plate. This plate has a high atomic number and it is usually made of titanium (Ti).^(1)^ Titanium implants and titanium alloys have gained popularity for their favorable mechanical properties and high tissue compatibility.^2^, ^3^

When the plate is used in the skull, it might interfere with the path of the beam for radiotherapy; therefore, the dose distributions can be affected by the presence of the plate.^4^, ^5^ The presence of metal inhomogeneities with high atomic number in the path of photon or electron beams alters the dose distribution, and may produce regions of hot and cold spots.^6^, ^7^, ^8^, ^9^, ^10^, ^11^ The effect of titanium mesh on the dose distribution has been evaluated in several studies.^5^, ^8^, ^12^, ^13^, ^14^, ^15^, ^16^, ^17^ Gagnon and Cundiff^(8)^ did an experiment with a parallel plate chamber to determine the magnitude of the dose enhancement as a function of incident electron energy, thickness, and atomic number of metal. They concluded that irradiation of a tissue‐metal interface with 13 MeV to 20 MeV electrons leads to an increased dose to the tissue on the entrance side of the metal. The presence of a metal plate resulted in a dose enhancement from 6% to 50%, compared to the case with no metal. In this study it was suggested that the increase in dose can be eliminated by the addition of $1-2\;g/{\text{cm}}^{2}$ of a low‐Z material between tissue and metal.^(8)^

Mian et al.^(13)^ examined the effect of bone‐metal interface on the radiation dose for ${\;}^{60}\text{Co}$ gamma rays, and 6 MV and 25 MV photons. They concluded that, for ${\;}^{60}\text{Co}$, there is a 15% increase in dose to solid bone at the entrance side of the titanium dental implants. For higher energy X‐rays, the increase in dose is slightly lower than ${\;}^{60}\text{Co}$. This increase in dose falls off rapidly and becomes negligible at 1–2 mm distance from the bone–metal interface.

Patone et al.,^(5)^ using film dosimetry, determined the dosimetric impact of a 0.4 mm thick neurosurgical titanium mesh for 6 and 18 MV photon beams. The titanium mesh in all measurements was placed at the depth of 1.5 cm in the phantom, and the films were placed above the mesh and below the mesh and the depth of 5 cm from the surface of the phantom. They concluded that, in the region above the titanium mesh, there is an increase in dose of 7.1% for 6 MV photons and 4.9% for 18 MV photons. In the region beyond the titanium mesh, there is an average decrease in dose of 1.5% for 6 MV photons and 1.0% increase in dose for 18 MV photons. At 5 cm depth, there was a 2.2% decrease in dose for 6 MV photons and 0.6% decrease for 18 MV photons. Their main conclusion was that, for cranial irradiation with high‐energy photons, the dosimetric impact of a titanium mesh with 0.4 mm thickness is very small and there is no need to modify the treatment parameters. However, they did not perform any experiment for electron beam.

In this study, the effect of the neurosurgical titanium mesh on the radiation dose distribution for electron beams is evaluated. The simulation and dosimetry are performed with Monte Carlo (MC) simulation and film dosimetry. By MC method, the dose inside and around titanium pieces can be calculated with high accuracy. The percent depth dose (PDD) is obtained in presence of the titanium mesh, and it is compared with PDD of homogeneus medium with no titanium mesh.

The results of the MC simulation and experimental measurements on dose distribution are compared with each other in various electron energies.

## II. MATERIALS AND METHODS

### A. Measurements

**Figure 1:**
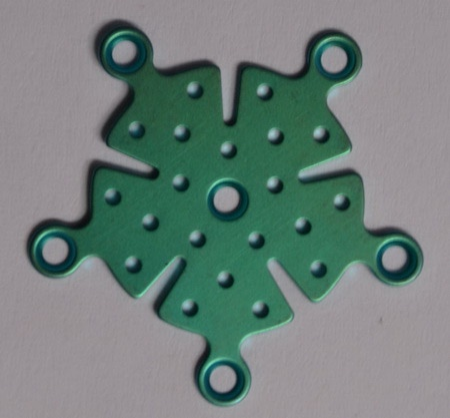
The image of the titanium mesh which is used for head and neck surgery. The outer length of the mesh is 25 mm and the thickness is 0.055 mm.

The titanium piece used in this study is a circular one, which is illustrated in Fig. 1 (titanium medium bone plate; General‐Implants, GmbH, Deutschland). The titanium thickness is 0.055 cm and the diameter of the plate is 2.5 cm. The plate is made of titanium alloy (Grade 5‐Ti‐6Al‐4V) with $4.42\;g/{\text{cm}}^{3}$ density. This kind of titanium mesh can be used in head and neck surgery.

Measurements were performed for 6 and 10 MeV electron beam on a 10PC Neptune medical linear accelerator dedicated for research purposes. The phantom used in the dosimetry consists of RW3 slabs. The RW3 phantom (PTW, Freiburg, Germany) with good approximation is water‐equivalent in the energy ranges from ${\;}^{60}\text{Co}$ to 25 MV photons and 4 MeV to 25 MeV electrons. The phantom slabs consist of one plate with 1 mm thickness, two plates of 2 mm thickness, one 5 mm plate, and 29 plates each with 10 mm thickness. This combination makes it possible to set the measurement depth in 1 mm steps. The size of the RW3 phantom was $30\;\text{cm}\times 30\;\text{cm}\times 20\;{\text{cm}}^{3}$. For film dosimetry, the GAFCHROMIC EBT2 radiochromic films were used. In this study, all of graphs and intensity maps have been plotted with an in‐house MATLAB software (MathWorks, Natick, MA).^(18)^

**Figure 2:**
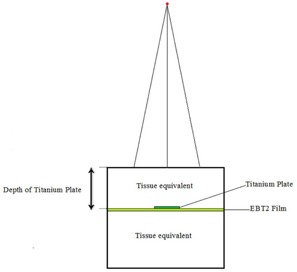
Experimental set up for measurement of the dose distribution using GAFCHROMIC films.

The experimental setup is shown in Fig. 2. In various measurements, the titanium mesh is placed at depths of 0.1, 0.5, 1, and 2 cm from the surface of the RW3 phantom. The EBT2 films were placed between the phantom slabs right after the titanium mesh. The size of the films was $5\times 5\;{\text{cm}}^{2}$. The films and the plate were placed along the central beam axis of the 6 MeV electron beam. The field size was $5\times 5\;{\text{cm}}^{2}$ and the phantom was placed on the couch at 100 cm source‐to‐surface distance. The above measurements were also repeated while the films were placed before the titanium mesh.

The same experiment for 10 MeV beam energy and titanium mesh depths of 0.5, 1, 2, 3, and 4 cm along the central beam axis was repeated.

Secondly, the same steps were performed without presence of the titanium mesh. In this case, the films were simply placed at the same depths between the slabs of the phantom and the same dose (same monitor unit) was delivered. The purpose of this experiment was the comparison of the absorbed dose of the film in presence of the titanium mesh and a homogeneous phantom.

EBT2 film calibration should be done as authoritative references advice to avoid unexpected errors. The films were calibrated and scanned according to Devic et al. recommendations.^(19)^ According to these recommendations each film is scanned five times before and five times after irradiation, and the average of the five scans is used for each case afterwards. The irradiated films in this study were scanned using Microtek ScanMaker 9800 pulse scanner (Microtek Lab Inc., Santa Fe Springs, CA). The scanned values for each pixel are converted to absorbed dose using the calibration curve. For calibration, the amount of radiation was changed from 5 cGy to 2000 cGy. The calibration is done by 6 MV photons with 1.5 cm slab on the film as a buildup. ^(19)^

After irradiation, the depth dose ratio with titanium and without titanium as a function of titanium mesh for various depths of the plate was obtained. The results are compared with values of Monte Carlo simulation in the following sections.

### B. Monte Carlo simulation

The simulations of Neptune 10PC medical linear accelerator (The Nepturn Laboratory, UCLA, Los Angeles, CA) is performed using BEAMnrc code. The DOSEXYZnrc code is used for phantom simulation and calculation of the absorbed dose in all of the phantom voxels. Phantom and radiation field size were designed similar to measurement setup. The MC geometry is similar to Fig. 2 with no EBT2 film. First, a homogeneous water phantom similar to measurement geometry was simulated. The size of voxels was $0.2\times 0.2\times 0.055\;{\text{cm}}^{3}$. For large depths, the thickness of the voxels was 0.5 and 1 cm, which makes the code faster for those voxels in electron field.

The absorbed dose in simulated phantom was measured at depths of 0.1, 0.5, 1, and 2 cm for 6 MeV and 0.5, 1, 2, 3, and 4 cm for 10 MV electrons along the central beam axis. Selected depths are similar to depths in the film dosimetry experiment. The cutoff energies for electrons (ECUT) and photons (PCUT) were set to 0.6 MeV and 0.01 MeV, respectively. In the next step, the water phantom with presence of titanium mesh was simulated. In this simulation, one titanium slab with the size of $2.2\times 2.2\times 0.055\;{\text{cm}}^{3}$ was placed in the center of water phantom perpendicular to the beam central axis. To partially simulate the holes on the titanium mesh, four voxels inside the titanium mesh were filled with air. The titanium mesh was placed at depths of 0.5, 1, 2, 3, and 4 cm similar to depths of measurements, and it was irradiated by 6 MeV and 10 MeV electron beam energies. In this case, for both 6 and 10 MV energies, the diagram of PDD as a function of titanium depth is obtained.

The value of dose in voxels right after titanium mesh is divided to the value of dose at same depth without presence of titanium mesh. This ratio gives the increasing of the dose due to the titanium mesh. The voxel is indicative of the tissue placed after the titanium mesh in contact with the plate. Diagram of dose ratio in this voxel, (ratio of dose with and without presence of titanium mesh) as a function of titanium depth at 6 and 10 MeV beam energies is acquired. Finally, the resulted numbers were compared to the same ratios obtained from film dosimetry.

## III. RESULTS & DISCUSSION

**Figure 3:**
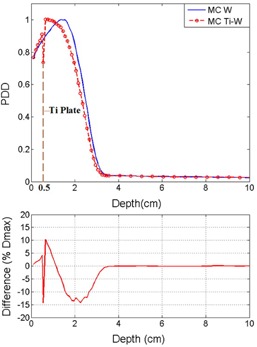
Comparison of MC results and PDD for two cases: with and without titanium mesh. The titanium mesh is placed at 0.5 cm depth and the electron beam energy is 6 MeV

**Figure 4:**
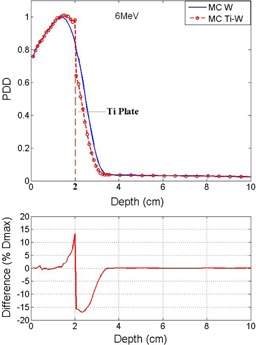
Comparison of MC results and PDD for 6 MeV electrons. The titanium mesh is this case is placed at depth of 2 cm, after Dmax.

Monte Carlo codes run with a number of histories in the order of 10^8^. Using MC simulation, the diagram of PDD for homogeneous phantom and the phantom with presence of the titanium mesh for 6 MeV electron beam energy are illustrated in Figs. 3 and Fig. 4. In Figs. 3 and 4, the depth of the titanium mesh is 0.5 cm and 2 cm, respectively, and the field size is $5\times 5\;{\text{cm}}^{2}$. The uncertainty of the doses in MC results were up to 2% in voxels with nonzero doses. The diagram of percentage of difference between the MC and measurement curves has also been inserted in the figures. The same graph was produced for various depths of the titanium mesh, which is not shown here, but the trend of these graphs is presented in Table 1.

Table 1 is related to data of MC simulation for the voxels immediately before and beyond the titanium mesh. The numbers in the table show the ratio of the dose of each voxel in presence of titanium mesh to the dose of that voxel in homogeneous phantom for various depths of the titanium mesh. The electron beam energy is 6 MeV.

**Figure 5:**
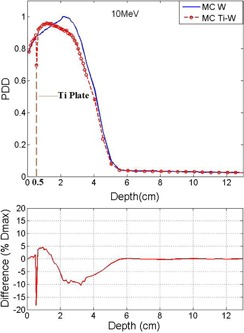
Comparison of PDD at two cases: with and without titanium mesh. The titanium mesh is placed in 0.5 cm depth and the electron beam energy is 10 MeV

**Figure 6:**
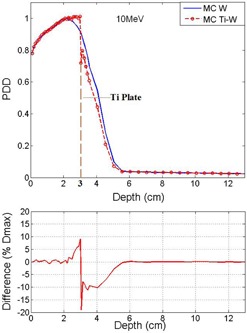
Comparison of MC results and PDD for 10 MeV electrons. The titanium plate is this case is placed at 3 cm, beyond Dmax.

Figures 5 and 6 compare the diagram of PDD as a function of depth in two phantoms with and without titanium mesh for 10 MeV beam energy. The titanium mesh is placed at 0.5 cm and 3 cm depth in Figs. 5 and 6, respectively.

In Table 2 the ratio of PDD in water phantom with and without titanium for 10 MeV electrons is given. The data are related to the voxels exactly beyond and before the titanium mesh. The numbers are derived from Monte Carlo calculation.

In the simulation, as illustrated in Figs. 3 to 6, inside the titanium the decreasing of the dose is observed. This is because of the lower mass collision stopping power of high‐Z materials, such as titanium and lead, with respect to water. The collisional stopping power results from coulomb interactions of electrons with orbital electrons of medium. This interaction results to energy transfer from incident electrons to medium.^(20)^

In all PDD results for the voxels before the titanium place there is always an increasing of the dose. This is because of backscattering of the titanium mesh. In comparison between Figs. 3 and 5 for shallow depths before the titanium, the relative amount of the backscattering for 6 MeV electrons is larger than 10 MeV electrons. The differences in voxels before the titanium are up to 4% and 2% for 6 and 10 MeV, respectively. That is because of reduced lateral and backscattering power for higher energy electrons. The same fact for the voxels before the titanium mesh was observed for photon energies in study of Patone et al.^(5)^ It should be noted that the technique of adding a low atomic number layer before the plate for reducing of backscattered electrons to the tissue is not feasible for titanium meshes. The modification of titanium mesh after the surgery is not possible.

**Table 1:** Differences in depth dose in water phantom with and without titanium (Ti) plate for the voxels before and beyond titanium mesh. The electron beam energy is 6 MeV and the data are from Monte Carlo simulations

*Beyond Ti*	*Before Ti*	*Ti Depth (cm)*
+9.3%	+2.1%	0.1
+10.3%	+4.2%	0.5
+8.2%	+9.5%	1
−16%	+13.4%	2

For the voxels beyond the titanium mesh considering the depth of the titanium and the energy of the beam, there is either increasing or decreasing of the dose. For the region before the depth of maximum dose (Dmax), there is an increasing of the dose and for the region beyond the Dmax, one has the decreasing of the dose. For the region before Dmax, the amount of increasing is up to 10% for both energies, as it is noted Tables 1 and 2. This behavior is observed for both 6 and 10 MeV electrons, and the ratios are less in 10 MeV electrons compare to 6 MeV.

**Figure 7:**
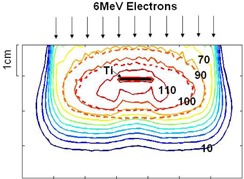
The isodose curves of 6 MeV electrons in presence of titanium mesh. The mesh is placed at 1 cm depth of the phantom. The numbers represent the percentage of the dose. The dashed lines represent the isodose curves for homogeneous phantom without the mesh.

An example of isodose curves calculated using MC in the plane perpendicular to the titanium mesh is illustrated in Fig. 7. In this figure, titanium plat is placed at 1 cm depth of the phantom and the incident beam is 6 MeV electrons. As it is illustrated, at the center of the mesh the isodose curves shrink toward the surface because of attenuation effect of the titanium. Near both edges of the mesh, hot spots are created in the depth dose. The amount of the hot spots were up to 112% and 105% for 6 MeV and 10 MeV beams, respectively.

**Table 2:** The ratio of the dose for the voxels before and beyond the titanium plate. The ratio is the amount of the dose for each voxel to the dose of the same voxel with the same depth in the homogeneous phantom. The electron beam energy is 10 MeV and the data are from Monte Carlo simulations

*Beyond Ti*	*Before Ti*	*Ti Depth (cm)*
+3.5%	+1.5%	0.5
+4.95%	+2.8%	1
+4.25%	+6.1%	2
−7.9%	+9.1%	3
−11.8%	+7.25%	4

In the case of an oblique surface, the obliquity increases the dose in shallower depths and decreases the dose in the deeper points.^(21)^ In the presence of the titanium mesh, the above effect (increasing and decreasing of the dose) would be strengthened.

**Figure 8:**
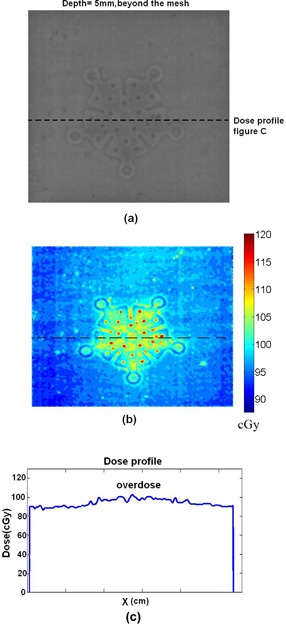
A sample (a) of the irradiated film for 6 MeV electrons for depth of 0.5 cm. The film is placed beyond the mesh. The dose (b) of the film in the colormap format using calibration curve. The dose profile (c) along the dashed line in (a).

**Figure 9:**
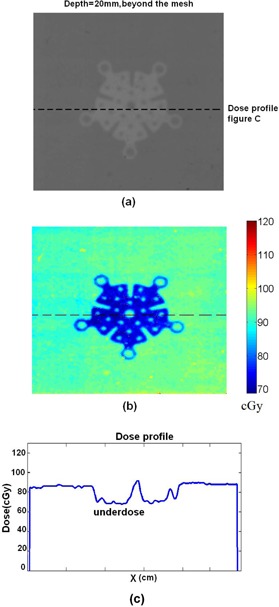
The irradiated film and the titanium plate in the depth of the 2 cm for 6 MeV electrons: (a) the irradiated film in gray scale; (b) the colormap of the dose; (c) the dose profile along the dashed line in (a).

**Figure 10:**
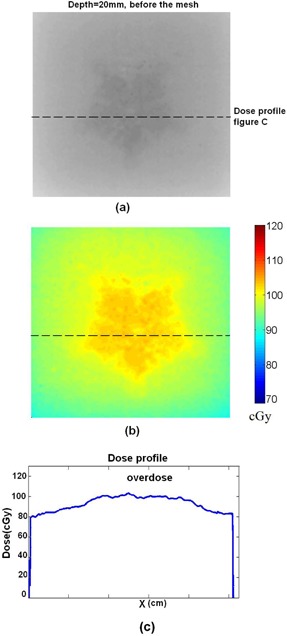
The results of dosimetry for a film which is placed “before” the titanium mesh at the depth of the 2 cm for 6 MeV electrons: (a) the irradiated film in gray scale; (b) the colormap of the dose; (c) the dose profile along the dashed line in (a).

For film dosimetry, the films are placed in various depths with titanium mesh on top. One of the films and its related dose in colormap is illustrated in Fig. 8(a) and (b). This film is related to the depth of 5 mm and it is placed beyond the mesh. Another film beyond the mesh with depth of 2 cm is illustrated in Fig. 9. In Fig. 8, one can observe the decreasing of the dose beyond the titanium mesh, and in Fig. 9 one can observe the decreasing of the dose compare with adjacent area around the titanium mesh. Figure 10 illustrates the results of the dose for the film which is placed before the titanium mesh at depth of 2 cm. The dose map and dose profile illustrate the overdose of adjacent tissue before the titanium mesh because of backscattering. In Fig. 10(b), the dose map, one can easily recognize the shape of the titanium mesh although the mesh is placed after the film. This shape is all created from increased backscattered electrons from the titanium mesh.

**Figure 11:**
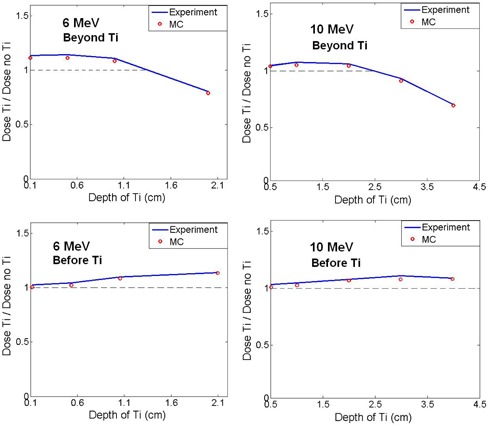
The ratio of dose with and without titanium plate in the voxel right before and beyond the titanium plate for various depth of the titanium plate. The experiment is the results of GAFCHROMIC film dosimetry. The energy of the beam is 6 and 10 MeV.

In the next step, the dose for the voxels right before and beyond the titanium mesh is evaluated. The value of the dose in voxel for homogeneous phantom is also calculated and the ratio of these two values are calculated and illustrated in Fig. 11. Therefore Fig. 11 illustrates the ratio of the dose before and beyond the titanium to the dose of that voxel without titanium for various depths of the titanium. The results are illustrated from Monte Carlo calculation, as well as film dosimetry. Figure 11(a) and (b) illustrate the results for 6 MeV and 10 MeV electrons, respectively. The results of experimental dosimetry are illustrated with a solid line, and the result of Monte Carlo is illustrated with red circles. The similar calculation is illustrated in Fig. 11 for the voxel before the mesh.

It should be noted that, for 6 MeV electrons, the maximum dose is at 1.5 cm depth (Dmax) and for 10 MeV, it is at 2.5 cm. As it is clear in Fig. 11(a) and (b), for both energies, the ratio of the dose before Dmax is larger than 1 and beyond Dmax it is less than 1. This means that, if the titanium mesh is placed in the buildup region, it will cause an overdose of the adjacent tissue, and if the titanium is placed beyond Dmax it leads to an underdose of the adjacent tissue. In Fig. 11, all the experiment data are a little larger than Monte Carlo results, which could be because of the small air gap outside the plate which is neglected in the Monte Carlo simulation.

## IV. CONCLUSIONS

In this study, using Monte Carlo simulation and film dosimetry, the effect of the titanium mesh on dose distribution is evaluated. This study is done for 6 and 10 MeV electrons. It was concluded that, for the voxels right beyond titanium, the change of the dose depends on the depth. If the titanium mesh is placed before depth maximum dose, Dmax, one has increasing of the dose for adjacent tissue and, if the titanium mesh is positioned in greater depths beyond maximum dose, it causes the underdose of the adjacent tissue. This fact is observed for both 6 and 10 MeV electrons.

For the voxels before the titanium mesh, there is always an increasing of the dose in compare to homogeneous cases. This conclusion is the same for 6 and 10 MeV energies in all depths of the titanium plat and is a result of the backscattering of the metal. In a realistic clinical case, titanium mesh are used in the superficial regions and most likely before Dmax. The fact has to be considered in dose calculation in the presence of titanium mesh.

## ACKNOWLEDGMENTS

Authors are grateful to neurosurgeon, Dr. Reza Pourkhalili, for providing useful information about the titanium mesh.
